# Pesticide application, educational treatment and infectious respiratory diseases: A mechanistic model with two impulsive controls

**DOI:** 10.1371/journal.pone.0243048

**Published:** 2020-12-03

**Authors:** Juan Pablo Gutiérrez-Jara, Fernando Córdova-Lepe, María Teresa Muñoz-Quezada, Gerardo Chowell

**Affiliations:** 1 Centro de Investigación de Estudios Avanzados del Maule (CIEAM), Universidad Católica del Maule, Talca, Región del Maule, Chile; 2 Facultad de Ciencias Básicas, Universidad Católica del Maule, Talca, Región del Maule, Chile; 3 Facultad de Ciencias de la Salud, Universidad Católica del Maule, Talca, Región del Maule, Chile; 4 School of Public Health, Georgia State University, Atlanta, Georgia, United States of America; Politecnico di Torino, ITALY

## Abstract

In this paper, we develop and analyze an SIS-type epidemiological-mathematical model of the interaction between pesticide use and infectious respiratory disease transmission for investigating the impact of pesticide intoxication on the spread of these types of diseases. We further investigate the role of educational treatment for appropriate pesticide use on the transmission dynamics. Two impulsive control events are proposed: pesticide use and educational treatment. From the proposed model, it was obtained that the rate of forgetfulness towards educational treatment is a determining factor for the reduction of intoxicated people, as well as for the reduction of costs associated with educational interventions. To get reduced intoxications, the population’s fraction to which is necessary to apply the educational treatment depends on its individual effectiveness level and the educational treatments’ forgetfulness rate. In addition, the turnover of agricultural workers plays a fundamental role in the dynamics of agrotoxic use, particularly in the application of educational treatment. For illustration, a flu-like disease with a basic reproductive number below the epidemic threshold of 1.0 is shown can acquire epidemic potential in a population at risk of pesticide exposure. Hence, our findings suggest that educational treatment targeting pesticide exposure is an effective tool to reduce the transmission rate of an infectious respiratory disease in a population exposed to the toxic substance.

## Introduction

Pesticides are chemical substances widely used in agriculture to control, prevent or eradicate pests of insects, birds, rodents, bacteria, herbs, among others [1]. Consequently, agricultural workers are frequently at risk of getting exposed to these toxic substances [2]. The effects of pesticide exposure in humans are varied, from vomiting, miosis, and respiratory problems to fetal malformations and cancer [3–5]. In particular, following pesticide poisoning, the respiratory tract dilates [6, 7], so it is plausible to establish that being in the condition of poisoning makes one more susceptible to infectious respiratory diseases. In this paper, we use mathematical modeling to investigate the transmission dynamics of respiratory infectious diseases after accounting for the link between pesticide exposure and increased susceptibility to acquiring the infection [8–13].

In the agricultural sector, pesticides use mostly occurs during the spring and summer seasons, with application at irregular intervals. Besides, prior studies have reported that individuals who become intoxicated from pesticides and seek healthcare are referred home by medical staff on duty, which lasts on average seven consecutive days with medical leave [14, 15].

To combat the negative effects of pesticides on human health, educational campaigns have demonstrated promising results [16–20]. During these campaigns, experts visit specific locations where these toxic substances are applied, particularly targeting agricultural workers to educate them on preventive measures to reduce the risk of becoming intoxicated with these substances. These measures include the correct implementation and use of protective equipment, the importance of handwashing, among others. These educational campaigns include applying questionnaires before and after the intervention, in order to assess whether the information was effectively assimilated in an integrated manner [21–23]. Studies recommend applying educational campaigns every two or three years (depending on the effectiveness) [24–26] to all subpopulations at risk of pesticide intoxication and not limited to agricultural workers [27, 28]. While these interventions help to reduce the rate of poisonings, their effectiveness could be further improved [29].

The application of educational campaigns for pesticide use as well as the treatment of poisoning cases requires significant resources. More specifically, the costs resulting from intoxications and infectious respiratory diseases include the costs associated with hospitalizations, medical licenses, and medicines [28, 30]. In addition, although educational treatments have an associated cost, the public health benefits they deliver are a relevant factor to consider.

While the dynamics of the variables involved in compartmental models of the spread of infectious-contagious respiratory are typically continuous over time, in our context, we require them to follow pulse-like temporal patterns because these are used, comparatively, at very narrow periods (discrete) of time. Thus the combined dynamics involve two different time scales: continuous (for example, disease transmission, the natural course of the disease) and discrete one (pesticide application periods). For this reason, it is appropriate to model these dynamics using a system of impulsive differential equations (IDE) [31], as these offer a convenient mathematical framework where the dynamics of some processes are characterized by abrupt changes in some instants of time, which are generally represented by pulses in the trajectory of their process, to model, for instance, the timing of harvests, abrupt changes in the stock market (crashes), among others [30, 32, 33]. The application of educational treatment is also intermittent and less frequent than the use of pesticides. Thus, this process can be modeled via a second sequence of impulsive events modeled via an IDE. Therefore, we have two scenarios that determine its respective sequences of pulse-times: (a) pesticide application and (b) educational campaigns.

In this study, we employ a compartmental impulsive susceptible-infectious-susceptible model (SIS-impulsive). From this model, we investigate the transmission dynamics of respiratory infectious diseases in the context of a population at risk of pesticide exposure since it induces enhanced susceptibility to infection. We incorporate educational campaigns that have the potential to prevent poisonings by pesticides.

In our analytic contribution, we derive threshold criteria (basic reproductive number type, BRN) in the context of our dynamic system of IDEs. It is worth noting that although there are techniques for calculating the BRN of an impulse system at fixed times [34–36], these are not directly applicable to our model that includes impulse duality, (a) and (b) [37].

In what follows, we gradually develop the mathematical model with the two pulse sequences (pesticide applications and educational interventions). Subsequently, we study the baseline dynamics of pesticides in the absence of infectious disease transmission and evaluate the associated costs. Next, we derive the threshold criteria using the theory associated with calculating the basic reproductive number. Moreover, we generate numerical simulations of the trajectories corresponding to the general model, including disease transmission dynamics. Finally, we discuss our interpretation of the results and suggest future directions for this work.

## Materials and methods

### Variables and model construction

#### Pesticide-free and untreated disease

The size of the susceptible and infectious groups of infectious respiratory disease and its interaction have been represented by the classic SIS model [38], with infection and recovery rates denoted by *β* and *γ*, respectively. It is important to mention that a frequency-dependent contact assumption is being used (the per capita contact rate between susceptible and infected individuals does not depend on the population density, so the transmission rate does not change with density). Generally speaking, this type of model applies to diseases such as bronchitis, acute respiratory infections, among others [9, 11, 12]. Neither birth rates nor mortality rates were considered, but rates of the entry (*b*) and exit (*d*) of agricultural workers to the system (study population).

#### Pesticide-using disease, but no educational treatment

In the context of pesticide use, we model a proportion (*μ*) of susceptible individuals that become intoxicated, leading to a new Susceptible-Intoxicated state that we denoted by *S*_*P*_. These susceptible individuals exhibit a higher probability of infection per contact with an infectious individual, when being exposed to the toxic substance. For this reason, we modeled two different transmission rates, the already mentioned *β* (without intoxication) and *β*_*P*_ (with intoxication), where *β* < *β*_*P*_.

On the other hand, as mentioned in the introduction, the application of pesticides is not applied steadily over time but rather over well-defined intervals of time, which we denoted by $t_{n}^{i}$, where *i* corresponds to the instants (days) in which the toxic substance is applied during the nth period (year), with $n,i\in Z^{+}$.

#### The disease with the use of pesticides and educational treatment: Double impulse

When educational treatment (campaigns) are incorporated in the model, the fraction of the population influenced by the intervention is denoted by *ϕ*. Furthermore, the compartments associated with the disease (*S*, *I*) must be subdivided into individuals targeted by education treatment campaigns (*S*_*T*_ and *I*_*T*_) and individuals who did not receive it (*S*_*N*_ and *I*_*N*_). In addition, in the subpopulation subject to educational campaigns (referred to here as Treaties), we further model the fraction of individuals that follow recommended protective behaviors to prevent toxic exposures to pesticides and those who do not. Besides, it is likely that individuals that undergo educational campaigns only follow protective behaviors for a limited period of time so that a fraction (*f*) of the “treated individuals” (treaties) become part of the group of Non-treaties. For this reason, it is necessary to carry out recurrent educational campaigns in the community where the timing of each intervention is denoted by *τ*_*n*_. It is worth mentioning that we will assume that these interventions are conducted before applying the substance toxic in the community.

#### Summary notation

The notation of the states is summarized in Table 1. Also, note that we do not incorporate an Infectious-Intoxicated state in the model because to be intoxicated does not affect the dynamics of transmission. Thus, our emphasis is on the effect of pesticide toxicity on susceptibility to the disease.

**Table 1:** Notation of model compartments.

	Susceptible	Infected
Treaties	*S*_*T*_	*I*_*T*_
Not treaties	*S*_*N*_	*I*_*N*_
Intoxicated	*S*_*P*_	-

Notation to be used throughout the text. Treaties are the individuals that effectively change their behavior following the educational campaign.

Similarly, the respective rates are summarized in Table 2 (the range of values will be used in the numerical simulations). It is worth mentioning that we will assume a closed and normalized population, i.e., the inflow (*b*) is equivalent to the outflow (*d*) and *N* = 1.

**Table 2:** Model parameter symbols, definitions and range of values used for numerical simulations.

Symbol	Meaning	Range	Reference
*β*	Infection of non-intoxicated individuals	0.3*	[38, 39]
*β*_*P*_	Infection of intoxicated individuals	0.65*	Author chosen
*γ*	Recovery rate	0.3*	[38, 39]
*b*	Entry rate	[0.001, 0.05]	Author chosen
*d*	Exit rate	[0.001, 0.05]	Author chosen
*μ*	Intoxication rate	[0.0043, 0.5]	[31, 40–42]
*ω*	Detoxification rate	[0.1, 0.5]	[31, 40–42]
*ϕ*	Treaties rate	[0, 1]	Author chosen
*f*	Forgetfulness rate	[0.00034, 0.01]	Author chosen

* Baseline value.

#### Flowchart and impulsive differential equation

**Fig 1:**
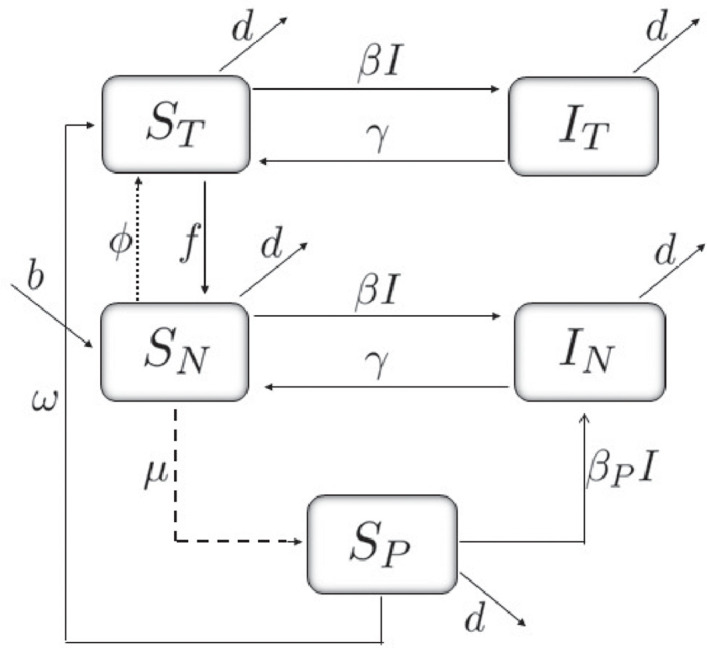
Dynamics of the states in which an individual can be found. *I* = *I*_*T*_ + *I*_*N*_. The segmented line corresponds to the pulse of the pesticide application and the dotted line to the pulse modeling the effects of the educational campaign.

The full schematic of our compartmental model diagram is displayed in Fig 1.

In the diagram (Fig 1), it can be seen that susceptibility is altered at the moment of pesticide application (segmented line), and infection may be acquired through the interaction of susceptible individuals with infectious individuals, either treated or untreated. In addition, the process that mitigates the effects of pesticide use in the population takes when the education campaigns are implemented (treatment) (dotted line), which induce positive health behavior change on susceptibles. Hence, susceptible individuals in the treated state (*S*_*T*_) exhibit a reduced risk of intoxication.

The mathematical model representing this interaction is given by the following system of impulsive differential equations:

$$\begin{array}{c} \left\{ \begin{array}{c} \begin{array}{cc} {\dot{S}}_{T} & =-\beta S_{T}I+\gamma I_{T}+\omega S_{P}-\left(d+f\right)S_{T}\\ {\dot{S}}_{N} & =-\beta S_{N}I+\gamma I_{N}+fS_{T}-dS_{N}+bN\\ {\dot{S}}_{P} & =-\beta_{P}S_{P}I-\left(\omega +d\right)S_{P}\\ {\dot{I}}_{T} & =+\beta S_{T}I-\left(\gamma +d\right)I_{T}\\ {\dot{I}}_{N} & =\left(\beta S_{N}+\beta_{P}S_{P}\right)I-\left(\gamma +d\right)I_{N} \end{array}\}\;t\notin \left\{t_{n}^{i},\tau_{n}\right\},\\ \begin{array}{cc} S_{T}\left(t^{+}\right) & =S_{T}\\ S_{N}\left(t^{+}\right) & =\left(1-\mu \right)S_{N}\\ S_{P}\left(t^{+}\right) & =S_{P}+\mu S_{N}\\ I_{T}\left(t^{+}\right) & =I_{T}\\ I_{N}\left(t^{+}\right) & =I_{N} \end{array}\}\;t=t_{n}^{i},\\ \begin{array}{cc} S_{T}\left(t^{+}\right) & =S_{T}+\phi S_{N}\\ S_{N}\left(t^{+}\right) & =\left(1-\phi \right)S_{N}\\ S_{P}\left(t^{+}\right) & =S_{P}\\ I_{T}\left(t^{+}\right) & =I_{T}\\ I_{N}\left(t^{+}\right) & =I_{N} \end{array}\}\;t=\tau_{n},\\ \tau_{n+1}=\tau_{n}+360 \end{array}\right. \end{array}$$ (1)

where *I* = *I*_*T*_ + *I*_*N*_. In the model, *n* represents the current year in which the toxic substance is applied and the educational protective campaign is conducted, while *i* denotes the time instants in which pesticides are applied.

### Dynamics of pesticides

The dynamics of pesticide use is an important initial baseline scenario to investigate in the absence of infectious disease transmission.

For the calculation of the infection-free solution, i.e., *I*_*T*_ = *I*_*N*_ = 0, the system (1) is reduced as follows:

$$\begin{array}{c} \left\{ \begin{array}{c} \begin{array}{cc} {\dot{S}}_{T} & =\omega S_{P}-\left(d+f\right)S_{T}\\ {\dot{S}}_{N} & =fS_{T}-dS_{N}+bN\\ {\dot{S}}_{P} & =-\left(\omega +d\right)S_{P} \end{array}\}\;t\notin \left\{t_{n}^{i},\tau_{n}\right\},\\ \begin{array}{cc} S_{T}\left(t^{+}\right) & =S_{T}\\ S_{N}\left(t^{+}\right) & =\left(1-\mu \right)S_{N}\\ S_{P}\left(t^{+}\right) & =S_{P}+\mu S_{N} \end{array}\}\;t=t_{n}^{i},\\ \begin{array}{cc} S_{T}\left(t^{+}\right) & =S_{T}+\phi S_{N}\\ S_{N}\left(t^{+}\right) & =\left(1-\phi \right)S_{N}\\ S_{P}\left(t^{+}\right) & =S_{P} \end{array}\}\;t=\tau_{n},\\ \tau_{n+1}=\tau_{n}+360. \end{array}\right. \end{array}$$ (2)

Thus, between the periods associated with pesticide applications, i.e. for any $t\in (t_{n}^{i},t_{n}^{i+1}]$, from the third equation of the system (2), one has to

$$\begin{array}{c} S_{P}\left(t\right)=S_{P}\left(t_{n}^{i^{+}}\right)e^{-\left(d+\omega \right)\left(t-t_{n}^{i^{+}}\right)}. \end{array}$$ (3)

In this way, it is reduced to finding the solution, written in matrix form,

$$\begin{array}{c} (\begin{array}{c} {\dot{S}}_{T}\\ {\dot{S}}_{N} \end{array})=(\begin{array}{cc} -(d+f) & 0\\ f & -d \end{array})(\begin{array}{c} S_{T}\\ S_{N} \end{array})+(\begin{array}{c} \omega S_{P}\left(t\right)\\ d \end{array}), \end{array}$$ (4)

with initial condition $\left(S_{T}\left(t_{n}^{i^{+}}\right),S_{N}\left(t_{n}^{i^{+}}\right)\right)$.

Then, the system solution (4) is given by

$$\begin{array}{c} (\begin{array}{c} S_{T}\\ S_{N} \end{array})=e^{A(t-t_{n}^{i^{+}})}(\begin{array}{c} S_{T}\left(t_{n}^{i^{+}}\right)\\ S_{N}\left(t_{n}^{i^{+}}\right) \end{array})+\int_{t_{n}^{i^{+}}}^{t}e^{A(t-s)}(\begin{array}{c} \omega S_{P}\left(s\right)\\ d \end{array})ds, \end{array}$$ (5)

where $A=(\begin{array}{cc} -(d+f) & 0\\ f & -d \end{array})$.

Therefore, the infection-free solution, for $t\in (t_{n}^{i},t_{n}^{i+1}]$, corresponds to (see S.1 Appendix)

$$\begin{array}{c} (\begin{array}{c} S_{T}\left(t\right)\\ S_{N}\left(t\right)\\ S_{P}\left(t\right) \end{array})=(\begin{array}{c} \left[S_{T}\left(t_{n}^{i^{+}}\right)+\frac{\omega }{\omega -f}S_{P}\left(t_{n}^{i^{+}}\right)\right]e^{-\left(d+f\right)\left(t-t_{n}^{i^{+}}\right)}-\frac{\omega }{\omega -f}S_{P}\left(t\right)\\ 1-\left[S_{T}\left(t_{n}^{i^{+}}\right)+\frac{\omega }{\omega -f}S_{P}\left(t_{n}^{i^{+}}\right)\right]e^{-\left(d+f\right)\left(t-t_{n}^{i^{+}}\right)}+\frac{f}{\omega -f}S_{P}\left(t\right)\\ S_{P}\left(t_{n}^{i^{+}}\right)e^{-\left(d+\omega \right)\left(t-t_{n}^{i^{+}}\right)} \end{array}). \end{array}$$ (6)

It is clearly observed that, in the absence of pulse instants, the system solutions converge (*t* → ∞) to a completely untreated population without intoxication (*S*_*N*_), as expected.

Note that the solution of state *S*_*T*_, for $t\in (t_{n}^{i},t_{n}^{i+1}]$, can be expressed in the form $S_{T}\left(t_{n}^{i^{+}}\right)e^{-\left(d+f\right)\left(t-t_{n}^{i^{+}}\right)}+\left[\omega /\left(\omega -f\right)\right]S_{P}\left(t_{n}^{i^{+}}\right)\left[e^{-\left(d+f\right)\left(t-t_{n}^{i^{+}}\right)}-e^{-\left(d+\omega \right)\left(t-t_{n}^{i^{+}}\right)}\right]$, so it can be inferred that this state at the time of pesticide application benefits from the treated population that exists at that time ($S_{T}\left(t_{n}^{i^{+}}\right)$) and after that, begins to decline through the rates of exit and oblivion (*d* and *f*). In addition, this dynamic is enhanced by a proportion *ω*/(*ω* − *f*) of individuals intoxicated at that instant ($S_{P}\left(t_{n}^{i^{+}}\right)$), who received self-care indications in a health care centre, by a difference (positive, so *ω* > *f*) between two exponential functions.

The behavior of the *S*_*N*_-state solution corresponds mainly to the difference between the total population and the *S*_*P*_-state solution, except for the fraction *f*/(*ω* − *f*) which is less than *ω*/(*ω* − *f*).

The third equation of the solution tells us that the Susceptible-Intoxicated state (*S*_*P*_) decreases exponentially based on the rates of exit and detoxification (*d* y *ω*), and has increasing impulses when the toxic substance is applied. Finally, the sum of the three solutions is one.

During the whole period of the nth year of pesticide application and considering the educational treatment at the beginning of the same year, the (*S*_*T*_, *S*_*N*_, *S*_*P*_) solution is given by (see S.2 Appendix):

$$\begin{array}{c} (\begin{array}{c} S_{T}\left(\tau_{n+1}\right)\\ S_{N}\left(\tau_{n+1}\right)\\ S_{P}\left(\tau_{n+1}\right) \end{array})=B_{t_{f}}B_{t_{p}}^{x-1}C_{t_{s}}(\begin{array}{c} S_{T}\left(\tau_{n}\right)\\ S_{N}\left(\tau_{n}\right)\\ S_{P}\left(\tau_{n}\right) \end{array}), \end{array}$$ (7)

where *x* corresponds to the number of pesticide applications during the nth year, plus *t*_*s*_, *t*_*p*_ and *t*_*f*_ represent the periods of application between treatment and pesticides, pesticide and pesticide, and finally pesticide and treatment respectively, i.e. $t_{s}=\left(\tau_{n},t_{n}^{1}\right]$, $t_{p}=\left(t_{n}^{l},t_{n}^{l+1}\right]$ and $t_{f}=\left(t_{n}^{x},\tau_{n+1}\right]$, with *l* ∈ {1, 2, …, *x* − 1}. The matrices described correspond to

$$\begin{array}{c} B_{t_{j}}=(\begin{array}{ccc} E_{f}\left(t_{j}\right) & \frac{\mu \omega }{\omega -f}\left[E_{f}\left(t_{j}\right)-E_{\omega }\left(t_{j}\right)\right] & \frac{\omega }{\omega -f}\left[E_{f}\left(t_{j}\right)-E_{\omega }\left(t_{j}\right)\right]\\ 1-E_{f}\left(t_{j}\right) & \mu (1-\frac{\omega }{\omega -f}\left[E_{f}\left(t_{j}\right)-E_{\omega }\left(t_{j}\right)\right]) & 1-\frac{\omega }{\omega -f}\left[E_{f}\left(t_{j}\right)-E_{\omega }\left(t_{j}\right)\right]\\ 0 & \mu E_{\omega }\left(t_{j}\right) & E_{\omega }\left(t_{j}\right) \end{array}), \end{array}$$

$$\begin{array}{c} C_{t_{s}}=(\begin{array}{ccc} E_{f}\left(t_{s}\right) & \phi E_{f}\left(t_{s}\right) & \frac{\omega }{\omega -f}\left[E_{f}\left(t_{s}\right)-E_{\omega }\left(t_{s}\right)\right]\\ 1-E_{f}\left(t_{s}\right) & \phi (1-E_{f}\left(t_{s}\right)) & 1-\frac{\omega }{\omega -f}\left[E_{f}\left(t_{s}\right)-E_{\omega }\left(t_{s}\right)\right]\\ 0 & 0 & E_{\omega }\left(t_{s}\right) \end{array}), \end{array}$$

with *t*_*j*_ ∈ {*t*_*p*_, *t*_*f*_} y $E_{m}\left(t_{j}\right)=e^{-\left(d+f\right)t_{j}}$, *m* ∈ {*f*, *ω*}.

Therefore from (7), it is concluded that the (*S*_*T*_, *S*_*N*_, *S*_*P*_) infection-free solution at the k-year-end is

$$\begin{array}{c} D^{k}(\begin{array}{c} S_{T}\left(\tau_{1}\right)\\ S_{N}\left(\tau_{1}\right)\\ S_{P}\left(\tau_{1}\right) \end{array}), \end{array}$$ (8)

where $D=B_{t_{f}}B_{t_{p}}^{x-1}C_{t_{s}}$

### Health costs

The poisonings by pesticides have associated costs (*C*_*P*_), within these can be expressed: (a) the costs that we denote *C*_*e*_, related (among others) to events such as medical licenses and personnel replacement, and (b) the costs associated with the hospital price (denoted by *C*_*h*_), as bed days and medicines. So, we can obtain that the total cost determined by the annual poisonings is:

$$\begin{array}{c} C_{P}=C_{e}\sum_{i=1}^{x}\left[S_{P}\left(t_{n}^{i^{+}}\right)-S_{P}\left(t_{n}^{i}\right)\right]+\left(C_{e}+C_{h}\right)\int_{\tau_{n}}^{\tau_{n+1}}S_{P}\left(t\right)dt, \end{array}$$ (9)

so (9) can be rewritten by:

$$\begin{array}{c} C_{P}=C_{e}\;\mu \sum_{i=1}^{x}S_{N}\left(t_{n}^{i}\right)+\left(C_{e}+C_{h}\right)\int_{\tau_{n}}^{\tau_{n+1}}S_{P}\left(t\right)dt. \end{array}$$ (10)

Resolving the integral defined in the period of one year, (10) gives:

$$\begin{array}{c} C_{P}=C_{e}\;\mu \sum_{i=1}^{x}S_{N}\left(t_{n}^{i}\right)+\frac{\left(C_{e}+C_{h}\right)}{d+\omega }\sum_{i=0}^{x}S_{P}\left(t_{n}^{i^{+}}\right)[1-e^{-\left(d+\omega \right)\left(t_{n}^{i+1}-t_{n}^{i^{+}}\right)}], \end{array}$$ (11)

where $t_{n}^{0^{+}}=\tau_{n}^{+}$ and $t_{n}^{x+1}=\tau_{n+1}$.

Let us observe that the cost can be controlled utilizing the rates *μ*, *d*, and *ω*. Hence, it is totally reasonable to think that if less toxic pesticides are applied, e.g., reducing *μ*, the quantity of intoxicated individuals decreases. Yet, that reasoning is not entirely correct, because the population is likely less concerned with low toxicity pesticides. Therefore, it is not enough to apply low toxicity pesticides for this measure to be effective. It must go hand in hand with educational interventions in order to guide and alert citizens about the consequences they may have on their health.

Educational treatment also has an associated cost (*C*_*T*_). These can be divided into fixed costs (*C*_*f*_) related to the expense of the expert personnel who will apply the treatment and the variable costs (*C*_*v*_), associated with the fraction of the population to which the instrument was applied. These can be expressed (annually) by the following equation:

$$\begin{array}{c} C_{T}=C_{f}+C_{v}\left[S_{N}\left(\tau_{n}^{+}\right)-S_{N}\left(\tau_{n}\right)\right], \end{array}$$ (12)

which can be rewritten by:

$$\begin{array}{c} C_{T}=C_{f}+C_{v}\;\phi \;S_{N}\left(\tau_{n}\right). \end{array}$$ (13)

Therefore the total annual cost (*C*_*Y*_) associated with pesticides, both for poisonings and educational treatment, is given by *C*_*Y*_ = *C*_*P*_+ *C*_*T*_, which can be reduced by privileging a correct and effective delivery of educational treatment over the fraction of the treated population.

The annual costs associated with pesticide intoxications (*C*_*P*_) from (11), associated with our model, these are altered by the rates of intoxication (*μ*), detoxification (*ω*), exit from system dynamics (*d*), costs associated with medical leave, staff replacement, among others (*C*_*e*_) and hospital costs (*C*_*h*_).

Based on previous studies [14, 43, 44], we have considered the following values in American dollars (USD) for simulation purposes in this section: *C*_*e*_ = 560 and *C*_*h*_ = 3000. With regard to the rates associated with intoxication (*μ* and *ω*), we have considered *μ* = 0.0043 and *ω* = 0.1 values in order to present projections in accordance with reality. In addition, regarding the departure fee (*d*) the value chosen is *d* = 0.001, so we can highlight the importance of the other fees already mentioned.

The costs associated with the educational treatment (*C*_*T*_), from (13) associated with our model, we can see that this value is affected by fixed costs (*C*_*f*_) and variable costs (*C*_*v*_), in addition to the fraction of the population that has received educational treatment (*ϕ*). The values assigned for these costs (USD) are *C*_*f*_ = 10000 and *C*_*v*_ = 100, which have been proposed as estimates in accordance with reality.

The total costs (*C*_*P*_ + *C*_*T*_) are expressed in the period of three years with an application of the educational treatment every two years, a frequency recommended by experts [24–26]. Thus, the educational treatment was applied at the beginning of the first and third simulated year. Pesticide applications were considered every ten days for a period of six months, with a total of 18 applications.

## Results

The numerical simulations shown below throughout the text were performed using MATLAB (The Mathworks, Inc). Values have been selected (see Table 2), mostly from the literature, in order to make the results more applicable. The rates have units of measurement corresponding to days^−1^. Concerning the selected values of *β* and *γ*, which correspond to the rates associated with the disease, and *d* to the rate of exit from the system, they were selected so that the basic reproductive number of the disease without the presence of pesticides, that is, the classic basic reproductive number of an SIS ($R_{0}^{c}=\beta /\left(\gamma +d\right)$) model is less than one [38, 45]. Therefore, since 1/*γ* represents the average time that an individual remains infectious, the assigned value expresses that an individual remains infectious between 3 and 4 days. Moreover, since the entry and exit rates to the dynamics delivered by the system are equal (*b* = *d*), there is a constant population. Because we want to explore the impact that pesticide poisoning has on the development of this type of disease, we have assigned the value of *β*_*P*_ greater than that of *β*. Moreover, in order to graphically show that the basic reproductive number can be greater than one in a population exposed to pesticides, high rates of intoxication have been selected (*μ*’s). Regarding the detoxification rate, this will depend on different factors, among which the types of pesticides stand out, such as Organophosphate, which takes between 24 and 48 hours to leave the body, while Diazinon takes around 12 days [40–42]. With regard to the fraction of the population to be treated (*ϕ*), this value varies from a population without treatment to a completely treated one. The forgetfulness rates were assumed to be similar to reality, so they were chosen in a range between 1% daily and monthly.

### Pesticide dynamics

**Fig 2:**
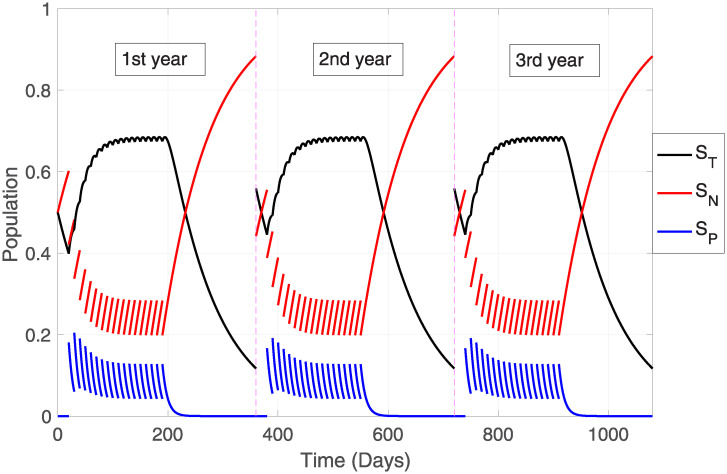
Model system trajectories in the absence of infectious disease transmission. General dynamics of pesticide application over three years with three educational interventions, each at the beginning of each year. *μ* = 0.3, *ω* = 0.1, *ϕ* = 0.5, *f* = 0.00134.

Obtained through numerical simulations, Fig 2, using the baseline parameter values (see Table 2), shows the trajectory of the system solutions (2), over a period of three years (considering a year equivalent to 360 days) with three applications of educational treatments before the annual use of pesticides (one for each beginning of the year of application of the toxic substance). These toxicants are applied every ten days for the first six months of each year.

**Fig 3:**
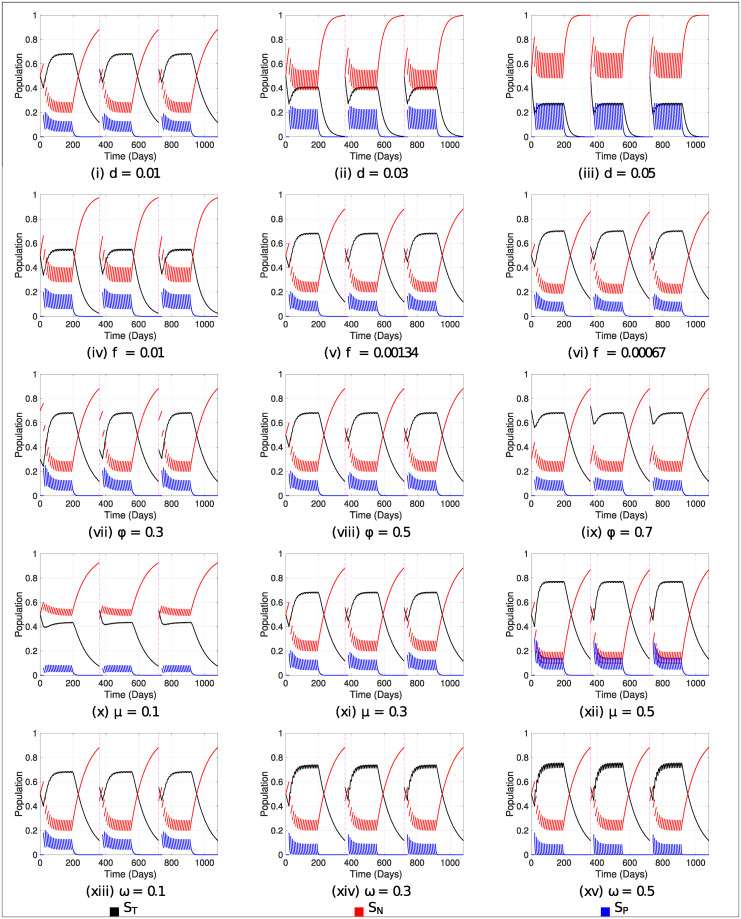
Variation in the parameters. The standard values used are *μ* = 0.3, *ω* = 0.1, *ϕ* = 0.5 and *f* = 0.00134. The variation of each of these parameters is presented under each figure, keeping the other three fixed.

Based on Fig 2, different dynamics are presented regarding the variation of parameters (exit rate, oblivion rate, treated population fraction, intoxication and detoxification rates) in order to visualize the effect of these indicators on the trajectories (see Fig 3).

In Fig 3(i)–3(iii) we can see that the greater the variability of individuals (agricultural workers), the more frequently educational treatment must be applied to a greater proportion of the population in order to be effective. In reality, this phenomenon of entry and exit to the agricultural population depends on the type of work they perform. It can also be seen that the greater the change in the variability of workers, the treated curve may be smaller than the curve of intoxicated individuals.

Regarding the forgetting rate, from 1% weekly (*f* = 0.00134), the trajectory of the treaties (*S*_*T*_) tends not to vary significantly, and at a lower rate, i.e. under 1% weekly (as in the case of 1% daily, i.e. *f* = 0.01), there is a difference between the different possible values (see Fig 3(iv)–3(vi))

The variation of the treated fraction (*ϕ*) overtime is not significant enough to establish the prolongation of group *S*_*T*_ (see Fig 3(vii)–3(ix)), so from Fig 3(iv)–3(vi), we can establish that, among the variables forgetting rate and fraction of the treated population, it is preferable to opt for an educational treatment applied with dedication and rigor (quality) over a greater number of people to whom the instrument is applied.

There is a wide variety of pesticides, hence a diversity of toxicity levels [46]. This is why it is necessary to understand the dynamics of the system (2) after different values of *μ*. We can infer that (see Fig 3(x)–3(xii)) while the pesticide is more toxic, educational interventions have a greater positive effect on the population exposed to the chemical; that is, there is less care for pesticides of lesser toxicity, leading to a greater number of cases of poisonings [21]. This is evident in our model since we assume that intoxicated individuals who resort to a health center also receive indications that are part of the treatment.

Given the wide variety of pesticides with different toxicity levels, there is also variation in recovery to the toxic substance, either because of its health status or directly because of the toxicity of the pesticide [47]. Thus, it is observed (see Fig 3(xiii)–3(xv)) that the variation of the recovery rate towards the pesticide (*ω*) affects the trajectories of the *S*_*T*_ and *S*_*P*_ states, since at a larger recovery scale, the intoxicated-curve can become null in several instants, and for the treated-curve, it tends to have impulsive events with more variability.

### General model dynamics

The basic reproductive number (BRN) of the pulseless system ($R_{0}^{c}$), corresponds to a representation of the classic BRN of a SIS system, this is

$$\begin{array}{c} R_{0}^{c}=\frac{\beta }{\gamma +d} \end{array}$$

Each variable exposed in the model affects the temporal trajectory of the epidemics. For numerical illustration, we will place ourselves in a context in which an infectious respiratory disease has a BRN lower than one (with an infected population corresponding to 1%), and show how this value is altered by the application of pesticides during the three-year period (a year will be considered equivalent to 360 days, as each month will be assumed to be 30 days). This is why we will focus on the sensitivity of the model to variables directly related to pesticide use and prevention: educational treatment and forgetting rate.

As mentioned in the introduction, the use of pesticides is centered in greater quantity between spring and summer, that is to say, during six months (180 days); based on this, we will assume for simulation effect that the toxic substance is applied every 10 days. Thus a total of 18 applications per year will be made, and in relation to the application of the educational treatment, we will assume it before the use of these toxics. In addition, to show the effect of the treatment in more detail, the first year without educational treatment will be simulated, and the other two years with treatment.

**Fig 4:**
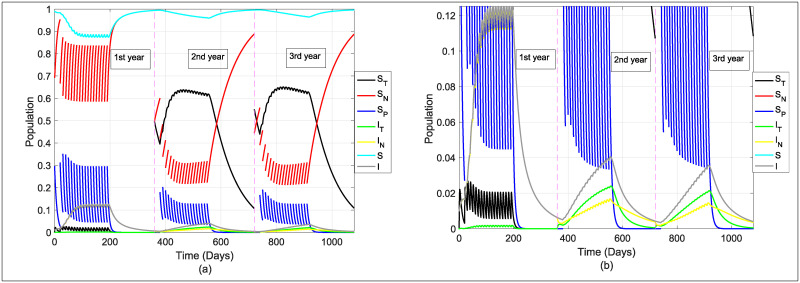
General model dynamics. First-year without the application of educational treatment and this leads to a higher number of infectious in that period. From the second year, the treatment is applied, decreasing the number of intoxicated and the number of infected. *S* = *S*_*T*_ + *S*_*N*_ + *S*_*P*_ and *I* = *I*_*T*_ + *I*_*N*_. Fig (a) Stander and Fig (b) with zoom. Used values: *β* = 0.3, *β*_*P*_ = 0.06, *γ* = 0.3, *b* = *d* = 0.01, *μ* = 0.3, *ω* = 0.1, *ϕ* = 0.5 and *f* = 0.00134.

The general dynamics of the model will be simulated under a context in which treatment is applied to 50% of the population and a forgetting rate corresponding to 1% weekly, this can be seen in Fig 4.

Note that in the first year (without treatment), the infectious curve is higher in relation to the curve of the following two years (with treatment). It is worth mentioning that during the first year, it is being considered that the population does not apply the recommendations given to them in public health services regarding exposure to these toxins, which is why the *S*_*T*_-state curve has small outbreaks that disappear quickly.

#### Forgetfulness rate sensitivity

**Fig 5:**
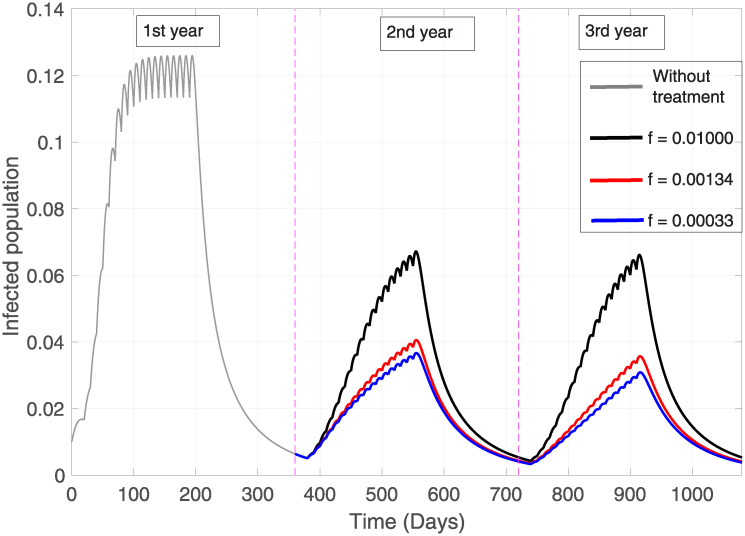
Infected population. Grey line without educational treatment. Black, red and blue line correspond to the forgetting rates of 1% daily, weekly and monthly respectively. *β* = 0.3, *β*_*P*_ = 0.65, *γ* = 0.3, *b* = *d* = 0.01, *μ* = 0.3, *ω* = 0.1, *ϕ* = 0.5

When applying educational treatment, it is important to study the effect it has on the “effective permanence” of the population, as there is a significant difference in the total number of infected cases including repetition (*A*_*T*_, the integral of the prevalence curve) after different rates of forgetfulness, fixing all the remaining parameters (see Table 3). Thus, if we establish a forgetfulness rate corresponding to 1% of the daily, weekly and monthly population, i.e. *f* = 0.01000, *f* = 0.00134 and *f* = 0.00034 respectively, it is equivalent to a total of infected with 19.9%, 15.4% and 14.7% respectively and approximately. So it is concluded that if the treatment is well accepted, understood, and applied by the community, this will be of great benefit not only for the reduction of intoxicated but also for the reduction of infected individuals (see Fig 5).

**Table 3:** Values assigned for numerical simulation.

	*A*_1_	*f*	*A*_2_	*A*_3_	*A*_*T*_
Value	9.6493	a) 0.01000	a) 5.1884	a) 5.0955	a) 19.9333
		b) 0.00134	b) 3.0780	b) 2.6628	b) 15.3901
		c) 0.00033	c) 2.7727	c) 2.2829	c) 14.7050

*A*_1_, *A*_2_ and *A*_3_ correspond to percentage of cumulative infectious cases of the first, second and third year respectively. *β* = 0.3, *β*_*P*_ = 0.65, *γ* = 0.3, *b* = *d* = 0.01, *μ* = 0.3, *ω* = 0.1, *ϕ* = 0.5.

It is important to reiterate that we are in a context where the disease without the presence of pesticide use has a lower BRN than one, so there is no epidemic. Thus, when entering the dynamics of pesticide use, the disease can become established. This is evidenced by the rise in proportion of population infection during the application of pesticides (first six months of each year, Fig 5), and when these toxic substances stop being applied (the last six months of each year) it can be seen that these epidemic curves decline before the pesticides are reapplied. Also, it is clearly observed (see Table 3) in the decrease of total infected between the first and second year of treatment.

#### Both variations

It is interesting to study how the infection curve is altered as a function of *ϕ* and *f*, as these are two parameters to be considered. If we look at Table 4, by not applying the educational treatment and being before a rate of forgetfulness corresponding to 1% daily, the infectious group amounts approximately to a total of 21% for two years. If treatment is applied to 40% of the exposed population, and this treatment is well acquired by citizens (so its oblivion rate decreases to 1% per month) the total infectious group with respect to the previous one decreases to 15%.

**Table 4:** Cumulative cases of infected (%) with respect to the variation of: forgetting rate and treatment.

Variation of the infectious group for two years			
Treatment	Forgotten rate of 1%		
	Daily (*f* = 0.01000)	Weekly (*f* = 0.00134)	Monthly (*f* = 0.00033)
*ϕ* = 0	20.5524	16.8881	16.3161
*ϕ* = 0.1	20.4482	16.6340	16.0389
*ϕ* = 0.2	20.3351	16.3580	15.7393
*ϕ* = 0.3	20.2122	16.0590	15.4168
*ϕ* = 0.4	20.0787	15.7363	15.0716
*ϕ* = 0.5	19.9333	15.3901	14.7050
*ϕ* = 0.6	19.7750	15.0216	14.3196
*ϕ* = 0.7	19.6027	14.6332	13.9195
*ϕ* = 0.8	19.4152	14.2286	13.5102
*ϕ* = 0.9	19.2114	13.8130	13.0984
*ϕ* = 1	18.9902	13.3928	12.6918

Cumulative cases of infectious (%) for two years in terms of forgetfulness and treatment rates. The rest of the rates are considered fixed with *β* = 0.3, *β*_*P*_ = 0.65, *γ* = 0.3, *d* = 0.01, *μ* = 0.3 and *ω* = 0.1.

**Fig 6:**
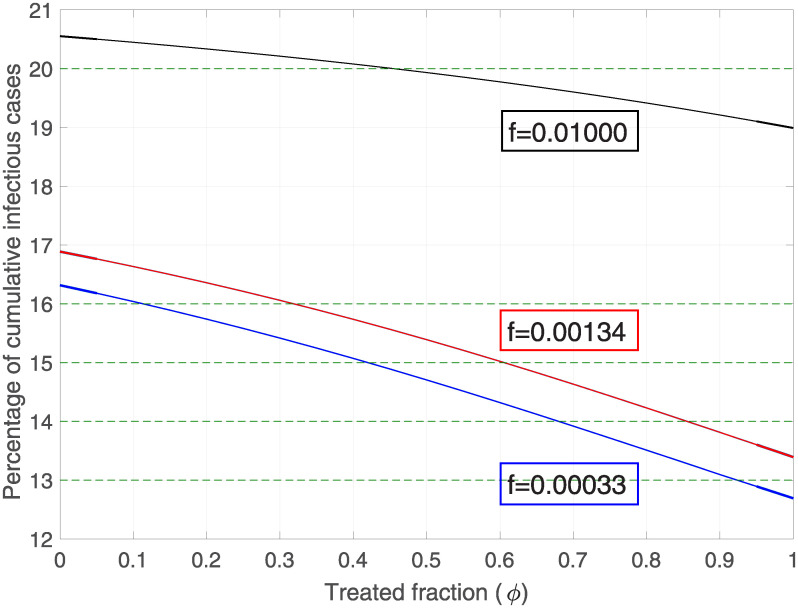
Cumulative infectious cases (%) v/s treated variation for different forgetting rates. Variation of the total percentage of infected people during the first two years with respect to the fraction of the population that has been treated. Black, red and blue lines correspond to the forgetting rates of 1% daily, weekly and monthly respectively. Used values: *β* = 0.3, *β*_*P*_ = 0.06, *γ* = 0.3, *b* = *d* = 0.01, *μ* = 0.3 and *ω* = 0.1.

Let us observe from Fig 6 that the total infected area is equivalent for different pairs of combinations with respect to the oblivion rates (*f*) and intervened population fraction (*ϕ*). For example, the cumulative cases of infectious corresponding to 15% is equivalent between 40% and 60% of the treated population, with an oblivion rate corresponding to 1% weekly and monthly, respectively. Therefore, there is a clear relationship between the quality of the intervention and the quantity treated.

There is an “extreme” case in the absence of educational campaigns (*f* = 1 and *ϕ* = 0). In this context, the total infected population is approximately 29%, which is much higher than the cases in which, although there is no educational treatment, there is an influence, either by health care centers, prevention campaigns, and/or the inspection of previous agricultural authorities; since this is evident among the different rates of forgetfulness (see row for values corresponding to *ϕ* = 0 in Table 4)

### Pesticide health costs

If we place ourselves in the case where educational treatment is not considered, i.e. *ϕ* = 0 and *f* = 1, the total cost for three years of pesticide application is USD 829380 (with an average annual cost of USD 276460), very close to the value presented in another study [43], since this study estimates an annual expenditure of USD 275920. In addition, if we look at Table 5, where *ϕ* = 0 the total costs vary, this is because although educational treatment is not being directly applied, in our model the warnings given in the health centres are being considered as part of the treatment. Let us observe that the effectiveness of the treatment (i.e. decreasing the rate of neglect, *f*) and the fraction of the population to which the treatment is applied (*ϕ*) are two considerable factors in reducing the costs associated with pesticide poisoning, i.e. incorporating monetary resources into educational treatment decreases the total cost (*C*_*P*_ + *C*_*T*_).

**Table 5:** Costs associated with intoxication and educational treatment for a period of three years.

Cost variation for three years (USD)			
Treatment	Forgotten rate of 1%		
	Daily (*f* = 0.01000)	Weekly (*f* = 0.00134)	Monthly (*f* = 0.00033)
*ϕ* = 0: Year 1	281170	278450	278000
Year 2	270590	259580	255510
Year 3	280570	266040	258350
**Total**	**832330**	**804070**	**791850**
*ϕ* = 0.1: Year 1	272220	258350	255670
Year 2	270410	251170	242130
Year 3	271670	244090	230970
**Total**	**814300**	**753620**	**728770**
*ϕ* = 0.2: Year 1	263269	2384240	233340
Year 2	270230	242770	228760
Year 3	262770	222780	205050
**Total**	**796270**	**703800**	**667160**
*ϕ* = 0.3: Year 1	254310	218140	211010
Year 2	270060	234370	215390
Year 3	253370	202110	180610
**Total**	**778240**	**654610**	**607010**
*ϕ* = 0.4: Year 1	245360	198030	188680
Year 2	269890	225970	202020
Year 3	244970	182070	157630
**Total**	**760210**	**606070**	**548330**
*ϕ* = 0.5: Year 1	236400	177930	166350
Year 2	269710	217570	188650
Year 3	236070	162670	136120
**Total**	**742180**	**558160**	**491110**
*ϕ* = 0.6: Year 1	227450	157820	144020
Year 2	269540	209160	175280
Year 3	227170	143910	116070
**Total**	**724150**	**510890**	**435370**
*ϕ* = 0.7: Year 1	218500	137720	121690
Year 2	269360	200760	161900
Year 3	218270	125780	97497
**Total**	**706130**	**464260**	**381090**
*ϕ* = 0.8: Year 1	209540	117610	99356
Year 2	269190	192360	148530
Year 3	209370	108290	80387
**Total**	**688100**	**418260**	**328280**
*ϕ* = 0.9: Year 1	200590	97504	77025
Year 2	269020	183960	135160
Year 3	200470	91440	64744
**Total**	**670080**	**372900**	**276930**
*ϕ* = 1: Year 1	191640	77399	54695
Year 2	268840	175550	121790
Year 3	191570	75226	50568
**Total**	**652050**	**328180**	**227050**

Cost variation (USD) for a period of three years in terms of forgetfulness and treatment rates. The rest of the rates are considered fixed with *μ* = 0.0043, *ω* = 0.1 y *d* = 0.001. The costs considered are *C*_*e*_ = 560, *C*_*h*_ = 3000, *C*_*f*_ = 10000 and *C*_*v*_ = 100.

**Fig 7:**
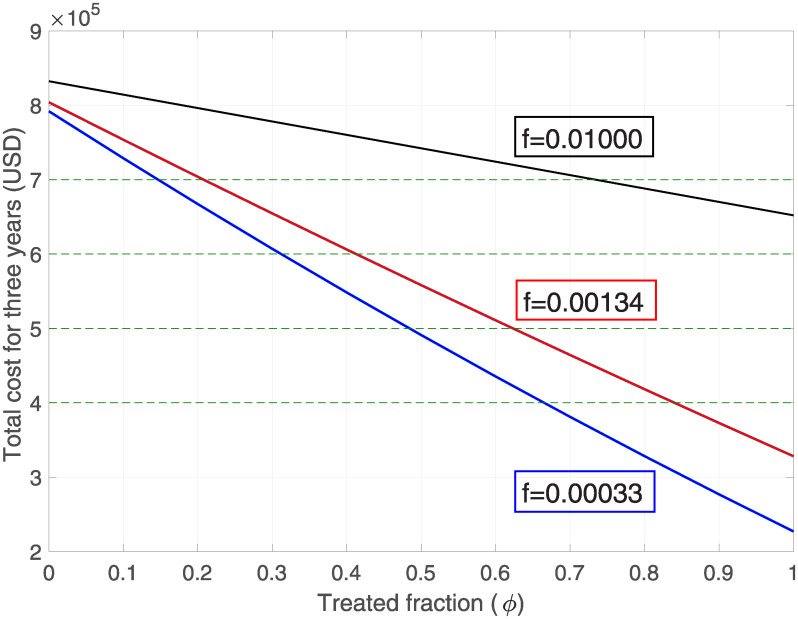
Total cost v/s treated variation for different forgetting rates. Variation of total cost during the three years with respect to the fraction of the population that has been treated. Black, red and blue lines correspond to the forgetting rates of 1% daily, weekly and monthly respectively.

In order to have a visual effect of the data displayed in Table 5, these values are expressed in Fig 7. It is explicitly observed how the decrease in the rate of forgetfulness establishes a more rapid decline when the fraction of the population that has received educational treatment increases.

## Discussion

The model’s main limitation is the generalist approach, concerning the disease and their respective associated rates. However, the aim is to present an infectious respiratory disease with a basic reproductive number lower than one and observe its behavior in a population exposed to pesticides, with data available from the associated intoxication and detoxification rates. Moreover, our analyses focuses on the costs associated with pesticide poisoning which do not include the costs associated with the infectious respiratory disease.

Through a system of differential equations with two pulses sequences, those associated with the application of pesticides and the educational treatment, we used the compartmentalized models’ methodology for analyzing these variables, supported by numerical simulations.

From mathematical modeling, there is only one previous work carried out by Gutiérrez et al. [8] that studies the dynamics between pesticide intoxications and infectious respiratory diseases, with an emphasis on the genetic susceptibility to the toxic substance, obtaining conclusions similar to ours regarding a possible spread, in a population exposed to pesticides, of an infectious disease that is under control.

The use of pesticides is concentrated in greater quantities during the spring-summer period [48, 49], while respiratory infections are concentrated in the autumn-winter period [50, 51]. However, in the regions of greatest use of these toxic substances poisoning occurs throughout the year [52]. The changes in the susceptibility of those in a poisoned condition alters the dynamics of respiratory infections, either by increasing the peaks of contagion, permitting new disease outbreaks, or prolonging the epidemic curve.

There is a diversity of interventions focused on reducing exposure or overexposure to pesticides, mainly aimed at agricultural workers [53]. These include (among others) methods of applying or mixing pesticides, using personal protection equipment for handling pesticides, educational campaigns, biological monitoring systems, pesticide alternatives, and pharmaceutical interventions [53–55]. In addition, there is environmental monitoring, such as passive air samplers [52], which provide tools for decision making and thus generate policies that enhance the control of air pollutants, such as pesticides that affect the respiratory tract. In our study, we have chosen the educational treatment (or campaigns) since they cover a large part of the content addressed among different types of interventions [16–19]. Furthermore, the educational campaigns are not only applied to agricultural workers but also educational establishments and the general population exposed to pesticides [20, 21, 31].

According to studies, educational treatment is recommended every two or three years (Appointments), which effectively reduces intoxication, but this frequency is not sufficient to reduce the chances of spreading infectious respiratory diseases that are controlled without the presence of these toxic substances.

The costs associated with pesticide intoxications vary among different countries, as do the resources available. From studies conducted [24–26], we can complement their recommendations that applying educational treatment every two years is feasible to decrease the costs associated with pesticide intoxications (including the cost per treatment). Findings on the health cost analyses are sensitive to the actual values of the cost variables. This is evident in the significant reduction in total health costs (associated with pesticides) as the fraction of the population treated increases or the rate of forgetfulness decreases (treatment efficacy). It is left for future work to include the costs associated with infectious respiratory diseases in order to visualize the impact that inclusion of such cost factors may have on our findings and public health decision-making.

The work carried out, leaves explicitly a route to follow, regarding the analytical calculation of the basic reproductive number for differential equation systems with two different pulse sequence involved. Furthermore, due to the shortage of models that present the dynamics between pesticide intoxications and infectious respiratory diseases, there is still a large number of epidemiological variables to be analyzed or incorporated into these dynamics, for example, types of exposure to pesticides, confusers associated with intoxication, among others.

## Conclusion

There are now public health policies to combat the morbidity and mortality burden associated with infectious respiratory diseases around the world. However, these public health policies aimed at mitigating transmission have not considered the role of pesticide poisonings and their consequences on public health in cities or communities where the great majority of the population are agricultural workers at risk of pesticide exposure [21]. However, there is research demonstrating the importance of prevention against pesticide exposure to reduce the number of poisonings significantly. Our work quantifies these conclusions from a mathematical modeling perspective, using an SIS-type model incorporating two instants of impulses, one for applying the toxic and the other for the implementation of educational treatment.

Our modeling findings underscore the possibility that a controlled infectious-contagious respiratory disease (BRN less than one) becomes epidemic (BRN greater than one) in a population with significant exposure to pesticides. Thus, educational treatment not only has the potential to mitigate intoxication rates but may also indirectly prevent sustained infectious disease transmission.

Intoxications and educational treatments have associated costs. One way to reduce the total costs (*C*_*Y*_) for pesticides, i.e., the sum of both components, is to prioritize the quality of the treatment above the fraction of the population treated. This preference leads to applying the educative intervention to a smaller fraction of the population over a more extended period.

It is tentative to apply low toxicity pesticides to reduce the intoxication significantly, which is a good measure in general. Nevertheless, the weakness or lack of consideration of this policy is that the citizenry commences less concerned about these toxics because they are less toxic. Therefore, the managers must accompany this measure with educational interventions [21].

## Appendix

### S.1. Infection-free solution for a period between pesticide applications

For *I*_*T*_ = *I*_*N*_ = 0, the system is reduced to:

$$\begin{array}{c} \left\{ \begin{array}{c} \begin{array}{cc} {\dot{S}}_{T} & =\omega S_{P}-\left(d+f\right)S_{T}\\ {\dot{S}}_{N} & =fS_{T}-dS_{N}+bN\\ {\dot{S}}_{P} & =-\left(\omega +d\right)S_{P} \end{array}\}\;t\notin \left\{t_{n}^{i},\tau_{n}\right\}\\ \begin{array}{cc} S_{T}\left(t^{+}\right) & =S_{T}\\ S_{N}\left(t^{+}\right) & =\left(1-\mu \right)S_{N}\\ S_{P}\left(t^{+}\right) & =S_{P}+\mu S_{N} \end{array}\}\;t=t_{n}^{i}\\ \begin{array}{cc} S_{T}\left(t^{+}\right) & =S_{T}+\phi S_{N}\\ S_{N}\left(t^{+}\right) & =\left(1-\phi \right)S_{N}\\ S_{P}\left(t^{+}\right) & =S_{P} \end{array}\}\;t=\tau_{n}\\ \tau_{n+1}=\tau_{n}+360 \end{array}\right. \end{array}$$ (14)

Thus, between the periods of pesticide applications, i.e. for any $t\in (t_{n}^{i},t_{n}^{i+1}]$, from the third equation of the system (14), one has to

$$\begin{array}{c} S_{P}\left(t\right)=S_{P}\left(t_{n}^{i^{+}}\right)e^{-\left(d+\omega \right)\left(t-t_{n}^{i^{+}}\right)} \end{array}$$ (15)

In this way, it is reduced to finding the solution, written in matrix form, of

$$\begin{array}{c} (\begin{array}{c} {\dot{S}}_{T}\\ {\dot{S}}_{N} \end{array})=(\begin{array}{cc} -(d+f) & 0\\ f & -d \end{array})(\begin{array}{c} S_{T}\\ S_{N} \end{array})+(\begin{array}{c} \omega S_{P}\left(t\right)\\ d \end{array}), \end{array}$$ (16)

with initial condition $\left(S_{T}\left(t_{n}^{i^{+}}\right),S_{N}\left(t_{n}^{i^{+}}\right)\right)$.

Then, the system solution (16) is given by

$$\begin{array}{c} (\begin{array}{c} S_{T}\\ S_{N} \end{array})=e^{A\;(t-t_{n}^{i^{+}})}(\begin{array}{c} S_{T}\left(t_{n}^{i^{+}}\right)\\ S_{N}\left(t_{n}^{i^{+}}\right) \end{array})+\int_{t_{n}^{i^{+}}}^{t}e^{A\;(t-s)}(\begin{array}{c} \omega S_{P}\left(s\right)\\ d \end{array})ds, \end{array}$$ (17)

where $A=(\begin{array}{cc} -(d+f) & 0\\ f & -d \end{array})$

The associated eigenvalues of matrix *A* are given by λ_1_ = −(*d* + *f*) and λ_2_ = −*d* whose eigenvectors are *v*_1_ = (*x*, −*x*) and *v*_2_ = (0, *y*), x,y∈R-{0} respectively. So *A* can be expressed in the form *A* = *PDP*^−1^, with $P=(\begin{array}{cc} x & 0\\ -x & y \end{array})$ and $P^{-1}=(\begin{array}{cc} 1/x & 0\\ 1/y & 1/y \end{array})$. For the sake of simplicity, the following shall be used $P=(\begin{array}{cc} 1 & 0\\ -1 & 1 \end{array})$ and $P^{-1}=(\begin{array}{cc} 1 & 0\\ 1 & 1 \end{array})$.

Thus, the exponential of the matrix can be expressed by:

$$\begin{array}{c} e^{A(t-t_{n}^{i^{+}})}=(\begin{array}{cc} e^{-\left(d+f\right)\left(t-t_{n}^{i^{+}}\right)} & 0\\ e^{-d(t-t_{n}^{i^{+}})}-e^{-\left(d+f\right)\left(t-t_{n}^{i^{+}}\right)} & e^{-d(t-t_{n}^{i^{+}})} \end{array}). \end{array}$$

Then

$$\begin{array}{c} e^{A\;(t-t_{n}^{i^{+}})}(\begin{array}{c} S_{T}\left(t_{n}^{i^{+}}\right)\\ S_{N}\left(t_{n}^{i^{+}}\right) \end{array})=(\begin{array}{c} S_{T}\left(t_{n}^{i^{+}}\right)e^{-\left(d+f\right)\left(t-t_{n}^{i^{+}}\right)}\\ e^{-d(t-t_{n}^{i^{+}})}[S_{T}\left(t_{n}^{i^{+}}\right)(1-e^{-f(t-t_{n}^{i^{+}}}+S_{N}\left(t_{n}^{i^{+}}\right)] \end{array}) \end{array}$$

and the integral $\int_{t_{n}^{i^{+}}}^{t}e^{A\;(t-s)}(\begin{array}{c} \omega S_{P}\left(s\right)\\ d \end{array})ds$, is equivalent to:

$$\begin{array}{c} \left(\begin{array}{c} \frac{\omega S_{P}\left(t_{n}^{i^{+}}\right)}{\omega -f}e^{-d(t-t_{n}^{i^{+}})}[e^{-f(t-t_{n}^{i^{+}})}-e^{-\omega (t-t_{n}^{i^{+}})}]\\ 1+\frac{f}{\omega -f}S_{P}\left(t_{n}^{i^{+}}\right)e^{-\left(d+\omega \right)\left(t-t_{n}^{i^{+}}\right)}+\frac{S_{P}\left(t_{n}^{i^{+}}\right)}{\omega -f}e^{-d(t-t_{n}^{i^{+}})}[\omega (1-e^{-f(t-t_{n}^{i^{+}})})-f-\frac{\left(\omega -f\right)}{S_{P}\left(t_{n}^{i^{+}}\right)}] \end{array}\right) \end{array}$$

So (16) can be expressed by

$$\begin{array}{c} (\begin{array}{c} S_{T}\left(t\right)\\ S_{N}\left(t\right) \end{array})=(\begin{array}{c} \left[S_{T}\left(t_{n}^{i^{+}}\right)+\frac{\omega }{\omega -f}S_{P}\left(t_{n}^{i^{+}}\right)\right]e^{-\left(d+f\right)\left(t-t_{n}^{i^{+}}\right)}-\frac{\omega }{\omega -f}S_{P}\left(t_{n}^{i^{+}}\right)e^{-\left(d+\omega \right)\left(t-t_{n}^{i^{+}}\right)}\\ 1-\left[S_{T}\left(t_{n}^{i^{+}}\right)+\frac{\omega }{\omega -f}S_{P}\left(t_{n}^{i^{+}}\right)\right]e^{-\left(d+f\right)\left(t-t_{n}^{i^{+}}\right)}+\frac{f}{\omega -f}S_{P}\left(t_{n}^{i^{+}}\right)e^{-\left(d+\omega \right)\left(t-t_{n}^{i^{+}}\right)} \end{array}). \end{array}$$ (18)

Therefore the free-infection solution for $t\in (t_{n}^{i},t_{n}^{i+1}]$, correspond to

$$\begin{array}{c} (\begin{array}{c} S_{T}\left(t\right)\\ S_{N}\left(t\right)\\ S_{P}\left(t\right) \end{array})=(\begin{array}{c} \left[S_{T}\left(t_{n}^{i^{+}}\right)+\frac{\omega }{\omega -f}S_{P}\left(t_{n}^{i^{+}}\right)\right]e^{-\left(d+f\right)\left(t-t_{n}^{i^{+}}\right)}-\frac{\omega }{\omega -f}S_{P}\left(t_{n}^{i^{+}}\right)e^{-\left(d+\omega \right)\left(t-t_{n}^{i^{+}}\right)}\\ 1-\left[S_{T}\left(t_{n}^{i^{+}}\right)+\frac{\omega }{\omega -f}S_{P}\left(t_{n}^{i^{+}}\right)\right]e^{-\left(d+f\right)\left(t-t_{n}^{i^{+}}\right)}+\frac{f}{\omega -f}S_{P}\left(t_{n}^{i^{+}}\right)e^{-\left(d+\omega \right)\left(t-t_{n}^{i^{+}}\right)}\\ S_{P}\left(t_{n}^{i^{+}}\right)e^{-\left(d+\omega \right)\left(t-t_{n}^{i^{+}}\right)} \end{array}). \end{array}$$ (19)

### S.2. Infection-free solution for a period between educational treatments

For the times between pesticide application, we must find the association between vectors $V_{n}^{i+1}=MV_{n}^{i}$, which are expressed by:

$$\begin{array}{c} (\begin{array}{c} S_{T}\left(t_{n}^{i+1}\right)\\ S_{N}\left(t_{n}^{i+1}\right)\\ S_{P}\left(t_{n}^{i+1}\right) \end{array})=M\left(t_{p}\right)(\begin{array}{c} S_{T}\left(t_{n}^{i^{+}}\right)\\ S_{N}\left(t_{n}^{i^{+}}\right)\\ S_{P}\left(t_{n}^{i^{+}}\right) \end{array}), \end{array}$$ (20)

where $M\left(t_{p}\right)=(\begin{array}{ccc} E_{f}\left(t_{p}\right) & 0 & \frac{\omega }{\omega -f}\left[E_{f}\left(t_{p}-E_{\omega }\left(t_{p}\right)\right)\right]\\ 1-E_{f}\left(t_{p}\right) & 0 & 1-\frac{\omega }{\omega -f}\left[E_{f}\left(t_{p}-E_{\omega }\left(t_{p}\right)\right)\right]\\ 0 & 0 & E_{\omega }\left(t_{p}\right) \end{array})$, $E_{f}\left(t_{p}\right)=e^{-\left(d+f\right)t_{p}}$ and $E_{\omega }\left(t_{p}\right)=e^{-\left(d+\omega \right)t_{p}}$.

Considering the respective impulse instants, from (20) you have equality

$$\begin{array}{c} (\begin{array}{c} S_{T}\left(t_{n}^{i+1}\right)\\ S_{N}\left(t_{n}^{i+1}\right)\\ S_{P}\left(t_{n}^{i+1}\right) \end{array})=M\left(t_{p}\right)(\begin{array}{ccc} 1 & 0 & 0\\ 0 & \left(1-\mu \right) & 0\\ 0 & \mu & 1 \end{array})(\begin{array}{c} S_{T}\left(t_{n}^{i}\right)\\ S_{N}\left(t_{n}^{i}\right)\\ S_{P}\left(t_{n}^{i}\right) \end{array}). \end{array}$$ (21)

So,

$$\begin{array}{c} (\begin{array}{c} S_{T}\left(t_{n}^{i+1}\right)\\ S_{N}\left(t_{n}^{i+1}\right)\\ S_{P}\left(t_{n}^{i+1}\right) \end{array})=B_{t_{p}}(\begin{array}{c} S_{T}\left(t_{n}^{i}\right)\\ S_{N}\left(t_{n}^{i}\right)\\ S_{P}\left(t_{n}^{i}\right) \end{array}), \end{array}$$ (22)

where

$$\begin{array}{c} B_{t_{p}}=(\begin{array}{ccc} E_{f}\left(t_{p}\right) & \frac{\mu \omega }{\omega -f}\left[E_{f}\left(t_{p}-E_{\omega }\left(t_{p}\right)\right)\right] & \frac{\omega }{\omega -f}\left[E_{f}\left(t_{p}-E_{\omega }\left(t_{p}\right)\right)\right]\\ 1-E_{f}\left(t_{p}\right) & \mu \{1-\frac{\omega }{\omega -f}\left[E_{f}\left(t_{p}-E_{\omega }\left(t_{p}\right)\right)\right]\} & 1-\frac{\omega }{\omega -f}\left[E_{f}\left(t_{p}-E_{\omega }\left(t_{p}\right)\right)\right]\\ 0 & \mu E_{\omega }\left(t_{p}\right) & E_{\omega }\left(t_{p}\right) \end{array}). \end{array}$$

Therefore, by recurrence from (22), we have to

$$\begin{array}{c} (\begin{array}{c} S_{T}\left(t_{n}^{i+1}\right)\\ S_{N}\left(t_{n}^{i+1}\right)\\ S_{P}\left(t_{n}^{i+1}\right) \end{array})=B_{t_{p}}^{x-1}(\begin{array}{c} S_{T}\left(t_{n}^{i}\right)\\ S_{N}\left(t_{n}^{i}\right)\\ S_{P}\left(t_{n}^{i}\right) \end{array}), \end{array}$$ (23)

What remains are the time periods between the application of the educational treatment and the first application of pesticides (*t*_*s*_) and the time period between the last application of pesticides and the next educational treatment (*t*_*f*_).

For *t*_*s*_, we have

$$\begin{array}{c} (\begin{array}{c} S_{T}\left(t_{n}^{1}\right)\\ S_{N}\left(t_{n}^{1}\right)\\ S_{P}\left(t_{n}^{1}\right) \end{array})=M\left(t_{s}\right)(\begin{array}{c} S_{T}\left(\tau_{n}^{+}\right)\\ S_{N}\left(\tau_{n}^{+}\right)\\ S_{P}\left(\tau_{n}^{+}\right) \end{array}), \end{array}$$ (24)

where $M\left(t_{s}\right)=(\begin{array}{ccc} E_{f}\left(t_{s}\right) & 0 & \frac{\omega }{\omega -f}\left[E_{f}\left(t_{s}-E_{\omega }\left(t_{s}\right)\right)\right]\\ 1-E_{f}\left(t_{s}\right) & 0 & 1-\frac{\omega }{\omega -f}\left[E_{f}\left(t_{s}-E_{\omega }\left(t_{s}\right)\right)\right]\\ 0 & 0 & E_{\omega }\left(t_{s}\right) \end{array})$, $E_{f}\left(t_{s}\right)=e^{-\left(d+f\right)t_{s}}$ and $E_{\omega }\left(t_{s}\right)=e^{-\left(d+\omega \right)t_{s}}$.

Considering the respective impulse instant, from (24) it follows that

$$\begin{array}{c} (\begin{array}{c} S_{T}\left(t_{n}^{1}\right)\\ S_{N}\left(t_{n}^{1}\right)\\ S_{P}\left(t_{n}^{1}\right) \end{array})=M\left(t_{s}\right)(\begin{array}{ccc} 1 & \phi & 0\\ 0 & \left(1-\phi \right) & 0\\ 0 & 0 & 1 \end{array})(\begin{array}{c} S_{T}\left(\tau_{n}\right)\\ S_{N}\left(\tau_{n}\right)\\ S_{P}\left(\tau_{n}\right) \end{array}). \end{array}$$ (25)

Similarly, for *t*_*f*_ it is obtained that

$$\begin{array}{c} (\begin{array}{c} S_{T}\left(\tau_{n+1}\right)\\ S_{N}\left(\tau_{n+1}\right)\\ S_{P}\left(\tau_{n+1}\right) \end{array})=M\left(t_{f}\right)(\begin{array}{ccc} 1 & 0 & 0\\ 0 & \left(1-\mu \right) & 0\\ 0 & \mu & 1 \end{array})(\begin{array}{c} S_{T}\left(t_{n}^{x}\right)\\ S_{N}\left(t_{n}^{x}\right)\\ S_{P}\left(t_{n}^{x}\right) \end{array}). \end{array}$$ (26)

Therefore, the infection-free solution between two pesticide applications (corresponding to one year) is given by:

$$\begin{array}{c} (\begin{array}{c} S_{T}\left(\tau_{n+1}\right)\\ S_{N}\left(\tau_{n+1}\right)\\ S_{P}\left(\tau_{n+1}\right) \end{array})=M\left(t_{f}\right)\;U\;B^{x-1}\;M\left(ts\right)\;H(\begin{array}{c} S_{T}\left(\tau_{n}\right)\\ S_{N}\left(\tau_{n}\right)\\ S_{P}\left(\tau_{n}\right) \end{array}), \end{array}$$ (27)

with $H=(\begin{array}{ccc} 1 & \phi & 0\\ 0 & \left(1-\phi \right) & 0\\ 0 & 0 & 1 \end{array})$ y $U=(\begin{array}{ccc} 1 & 0 & 0\\ 0 & \left(1-\mu \right) & 0\\ 0 & \mu & 1 \end{array})$.

Note that (27) can be rewritten as:

$$\begin{array}{c} (\begin{array}{c} S_{T}\left(\tau_{n+1}\right)\\ S_{N}\left(\tau_{n+1}\right)\\ S_{P}\left(\tau_{n+1}\right) \end{array})=B_{t_{f}}\;B_{t_{p}}^{x-1}\;C_{t_{s}}(\begin{array}{c} S_{T}\left(\tau_{n}\right)\\ S_{N}\left(\tau_{n}\right)\\ S_{P}\left(\tau_{n}\right) \end{array}), \end{array}$$ (28)

where $B_{t_{f}}=M\left(t_{f}\right)\;U$ y $C_{t_{s}}=M\left(t_{s}\right)\;H$.
